# Small extracellular vesicles from human umbilical cord mesenchymal stem cells delivering miR-202-5p alleviate renal ischemia-reperfusion injury by targeting the GOLIM4/PI3K/AKT axis

**DOI:** 10.3389/fimmu.2025.1586174

**Published:** 2025-05-30

**Authors:** Xiang Peng, Wei Shi, Haitao Yu, Zhenwei Feng, Zongjie Wei, Weiyang He, Xin Gou, Yongpeng Xie

**Affiliations:** ^1^ Department of Urology, The First Affiliated Hospital of Chongqing Medical University, Chongqing, China; ^2^ Chongqing Key Laboratory of Molecular Oncology and Epigenetics, Chongqing, China; ^3^ Center for Reproductive Medicine, Women and Children’s Hospital of Chongqing Medical University, Chongqing Health Center for Women and Children, Chongqing, China; ^4^ Department of Molecular and Medical Pharmacology, David Geffen School of Medicine, University of California, Los Angeles, Los Angeles, CA, United States

**Keywords:** renal ischemia reperfusion injury, acute kidney injury, human umbilical cord mesenchymal stem cells, small extracellular vesicles, miR-202-5p, GOLIM4

## Abstract

**Background:**

Ischemia–reperfusion injury (IRI) is a leading contributor to acute kidney injury (AKI), resulting in severe renal dysfunction and increased mortality. Despite progress in medical research, effective therapies for IRI remain limited. Recently, small extracellular vesicles (sEVs) originating from human umbilical cord mesenchymal stem cells (HucMSC-sEVs) have gained attention as potential therapeutic agents for alleviating organ damage. This study aimed to investigate the protective effects of HucMSC-sEVs in renal IRI and explore the underlying mechanisms involved.

**Methods:**

HucMSC-sEVs were isolated from HucMSCs via differential ultracentrifugation. Their characteristics were analyzed via transmission electron microscopy (TEM), nanoFCM, and western blotting. The protective effects of HucMSC-sEVs on OGD/R-induced apoptosis in HK-2 cells were evaluated via western blotting and flow cytometric analysis. Additionally, to explore the molecular mechanisms, qRT-PCR, dual-luciferase reporter assays, and other techniques were employed to investigate the role of miR-202-5p in HucMSC-sEVs, with a focus on its ability to regulate the PI3K/AKT pathway through the targeting of GOLIM4. Finally, the therapeutic effects of HucMSC-sEVs were evaluated *in vivo* via a mouse model of IRI.

**Results:**

The HucMSC-sEVs exhibited a characteristic biconcave circular morphology, with a particle size range of 60–100 nm and an average diameter of 79.8 nm. Western blotting confirmed the presence of sEV markers CD9 and TSG101, and HucMSC-sEVs were efficiently taken up by HK-2 cells. In the OGD/R model, HucMSC-sEVs significantly reduced apoptosis, attenuated the expression of BAX and CC3, and promoted the upregulation of BCL-2. Mechanistic studies revealed that HucMSC-sEVs deliver miR-202-5p, which targets GOLIM4 and activates the PI3K/AKT pathway, ultimately reducing renal tubular cell apoptosis. In the mouse IRI model, HucMSC-sEVs significantly alleviated kidney damage and reduced the serum creatinine and urea nitrogen levels.

**Conclusion:**

This study is the first to demonstrate the role of HucMSC-sEVs in attenuating renal IRI both *in vitro* and *in vivo* through the modulation of the GOLIM4/PI3K/AKT pathway via miR-202-5p. These findings identify a novel molecular target for the treatment of AKI via HucMSC-sEVs and provide a strong theoretical basis for their potential clinical application.

## 1 Introduction

Renal ischemia-reperfusion injury (IRI), a common and serious complication subsequent to surgical procedures, trauma, and organ transplantation, is characterized by the transient cessation of blood flow (ischemia) followed by the restoration of blood supply (reperfusion) (1–3). This phenomenon can initiate a cascade of pathophysiological events, including acute inflammatory responses, oxidative stress, and apoptosis (4). Renal IRI is regarded as a leading cause of acute kidney injury (AKI), and despite the lack of effective diagnostic tools, early detection of AKI remains a significant challenge (5). A significant number of individuals afflicted with AKI may develop chronic kidney disease or even reach end-stage renal disease in the absence of efficacious therapeutic interventions, which can profoundly compromise their long-term prognosis (1, 6, 7). Consequently, investigating efficacious approaches for the treatment and prevention of AKI resulting from IRI is highly important.

Apoptosis plays a central role in the pathology of renal IRI (8). The ischemic state and the subsequent reperfusion induce oxidative stress and energy imbalance within renal cells, thereby activating apoptotic pathways and exacerbating renal tubular cell death (9). Research indicates that apoptosis not only amplifies the extent of renal tissue damage but also initiates localized inflammatory responses, which further aggravate renal failure and facilitate the progression of AKI (10). Previous studies have shown that the inhibition of apoptosis is considered an effective approach to mitigate renal IRI. Modulating apoptosis-related signaling pathways, particularly by decreasing the expression of proapoptotic factors or increasing the levels of antiapoptotic proteins, can significantly alleviate renal injury and promote tissue repair and functional recovery (11–13). Therefore, the targeted inhibition of apoptosis may emerge as a crucial therapeutic strategy for renal IRI.

Human umbilical cord mesenchymal stem cells (HucMSCs) and their secreted small extracellular vesicles (HucMSC-sEVs) have demonstrated considerable therapeutic potential in alleviating IRI (14, 15). HucMSCs exhibit notable anti-inflammatory, antiapoptotic, and tissue repair-promoting properties, which are primarily attributed to the biological activities of their secreted HucMSC-sEVs (15–17). These extracellular vesicles, at the nanoscale, are rich in bioactive molecules, including proteins, lipids, and nucleic acids, that are instrumental in facilitating intercellular communication (18). Previous studies have shown that HucMSC-sEVs can mitigate the cellular damage caused by renal IRI and enhance tissue function by modulating inflammatory and apoptotic signaling pathways (12, 19). Furthermore, microRNAs (miRNAs), which are constituents of small extracellular vesicles (sEVs), have gained interest for their regulatory roles on IRI (20, 21). miRNAs are a category of noncoding RNA molecules approximately 22 nucleotides in length that primarily exert their regulatory effects by binding to the 3’ untranslated region (3’UTR) of target genes, thereby impeding their transcription or translation (22). In the context of IRI, numerous miRNAs have been shown to be closely associated with inflammatory responses, apoptotic processes, and oxidative stress (23). Consequently, investigating the mechanistic roles of HucMSC-sEVs and miRNAs in renal IRI could offer critical insights and facilitate the advancement of miRNA-based precision therapies.

Golgi integral membrane protein 4 (GOLIM4), a pivotal protein located on the Golgi membrane, is intricately involved in the processes of intracellular membrane trafficking and signal transduction (24). Furthermore, Lin et al. (25) reported that miR-105-3p promotes the proliferation and metastasis of breast cancer cells by targeting and inhibiting the expression of GOLIM4. However, research on its role in IRI remains limited. Concurrently, the phosphatidylinositol 3-kinase/protein kinase B (PI3K/AKT) signaling pathway, a key cell survival pathway, plays a critical role in modulating antiapoptotic processes, facilitating cell proliferation, and injury repair (26). Disruption of this pathway is deemed a pivotal element in the pathological progression of IRI (8, 27). Consequently, the interaction between GOLIM4 and the PI3K/AKT pathway, and their regulatory roles in renal IRI, is crucial for understanding disease etiology and guiding the development of novel therapeutic strategies.

Our study aimed to assess the therapeutic efficacy of miR-202-5p-enriched HucMSC-sEVs in the context of renal IRI, both *in vitro* and *in vivo*. We hypothesized that delivering miR-202-5p through HucMSC-sEVs could suppress GOLIM4 expression, subsequently modulating the PI3K/AKT signaling pathway and diminishing apoptosis in renal tubular cells. These findings suggest a potential therapeutic approach for alleviating renal IRI and offer a promising strategy for the clinical management of AKI.

## 2 Materials and methods

### 2.1 Cell culture and handling

Human renal proximal tubular epithelial cells (HK-2) and human umbilical cord mesenchymal stem cells (HucMSCs) were obtained from Procell (Wuhan, China) and cultured in DMEM/F12 medium (Gibco, USA) supplemented with 10% fetal bovine serum (FBS) (Gibco, USA) and 1% penicillin–streptomycin at 37°C a humidified incubator. In accordance with MISEV2023 of the Journal of Extracellular Vesicles (28), to ensure that the experimental results were not influenced by sEVs present in FBS, sEVs were depleted from FBS through ultracentrifugation at 140,000 × g in complete DMEM/F12 medium for 18 hours (Type 45 Ti rotor, k-factor 217.6, from Beckman Coulter, Brea, CA).

To simulate renal IRI *in vitro*, a chemical anoxia/recovery method, oxygen-glucose deprivation/reperfusion (OGD/R), was used. Initially, HK-2 cells were incubated in a hypoxic solution for 1 hour at 37°C and in 5% CO2. This solution contained a glucose-free medium, supplemented with antimycin A (5 μM) and 2 deoxy-D-glucose (5 mM). Subsequently, the hypoxic solution was replaced with complete medium, and the cells were cultured for an additional 24 hours.

A coculture model was established to assess the protective effect of sEVs derived from HucMSCs (HucMSC-sEVs) on HK-2 cells subjected to OGD. HucMSCs were cultured in Transwell inserts (0.4 µm, Corning, USA). After 48 hours, the inserts were transferred to six-well plates containing OGD-treated HK-2 cells and incubated for an additional 24 hours. To establish *in vitro* models, 30 μg HucMSC-sEVs were supplemented into the complete medium of 10^6^ HK-2 cells for 24 h.

### 2.2 Cell transfection

The sequences of the negative control, miR-202-5p mimic, miR-202-5p inhibitor, and siR-GOLIM4 were designed and synthesized by Tsingke (Beijing, China). Transfections were carried out according to the manufacturer’s instructions, using Lipofectamine 2000 or Lipofectamine 3000 (Thermo Fisher Scientific, USA). When HK-2 cells reached approximately 60% confluence, they were transfected with the miR-202-5p mimic or inhibitor using Lipofectamine 2000 for 6 hours. For siR-GOLIM4 transfection, Lipofectamine 3000 was used for 6 hours. After transfection, the cells were washed twice with PBS and then cultured in complete DMEM/F12 medium at 37°C with 5% CO2 for 24 hours.

### 2.3 sEVs separation and concentration

HucMSC cells were cultured in complete DMEM/F12 medium depleted of sEVs. Upon reaching 80-90% confluence, the cell density in the cell culture mediaum (CCM) was approximately 5×10^7 cells per 15-cm dish. Subsequently, the conditioned mediaum was harvested for EV separation. At the time of conditioned media harvest, the percentage of dead cells was approximately 5-10%.

SEVs were separated from CCM by differential ultracentrifugation as described (29), with minor modifications. Briefly, the CCM was processed through a series of centrifugation steps: 300 × g for 10 minutes, 2,000 × g for 20 minutes, and 12,000 × g for 30 minutes to remove cells and cellular debris. The resulting supernatants were then filtered through a 0.22 μm filter (Millipore) by gravity to remove larger debris. After ultracentrifugation at 140,000 × g for 70 minutes (using a Type 45 Ti rotor with a k-factor of 133, maximal acceleration, and deceleration, Beckman Coulter), the pellet was gently resuspended in sterile PBS. The suspension was then subjected to a second round of ultracentrifugation at 140,000 × g for 70 minutes to pellet sEVs, which were subsequently resuspended in 100 μl of sterile PBS for further analyses.

### 2.4 sEVs identification

Transmission electron microscopy (TEM, Hitachi-7500, Yokohama, Japan) was utilized to examine the morphology of sEVs. Briefly, 30 μl of sEVs samples were deposited onto a 100-mesh copper grid and allowed to air dry for 10 minutes, followed by drying with filter paper. Finally, the grid was stained with phosphotungstic acid for 15 seconds and left to dry at room temperature. NanoFCM was used to analyze the particle size distribution and concentration of sEVs. The purified sEVs samples were diluted to appropriate concentrations and subsequently analyzed using the laser scattering system of the instrument (N30E, NanoFCM). SEVs markers (CD9, TSG101) and a negative control protein (Calnexin) were detected by western blotting to identify the presence of sEVs.

### 2.5 sEVs internalization

*In vitro*, firstly, the isolated sEVs were labeled with the fluorescent dye PKH67 (Sigma-Aldrich) for 4 minutes, after which the incubation was terminated with complete medium. Subsequently, ultracentrifugation was performed for 70 minutes at 140,000 × g to remove any unbound dye. The labeled sEVs were then added to HK-2 cells in the logarithmic growth phase and incubated for 6 and 12 hours. At the end of these periods, the cytoskeleton was labeled with ActinRed (Invitrogen) and the nucleus with DAPI. *In vivo*, sEVs were labeled with PKH26 (Sigma-Aldrich), and then injected through the tail vein 1 hour prior to the establishment of the IR model. After 24 hours of reperfusion, the kidneys were excised and sectioned for freezing. The internalization of sEVs was observed both *in vitro* and *in vivo* using laser scanning confocal microscopy (Olympus Fluoview 2000).

### 2.6 Flow cytometric analysis

Initially, HK-2 cells from different treatment groups were treated with 0.25% trypsin at 37°C for 2 minutes. Following this, the cells were centrifuged at 1000 × g for 5 minutes to collect them. The cells were then resuspended in PBS and stained with propidium iodide and Annexin V-FITC (Beyotime, China). Finally, the flow cytometer (BD Biosciences, CA) was used to detect the proportion of apoptotic HK-2 cells in each treatment group.

### 2.7 Western blotting

Cells were homogenized in RIPA buffer (Beyotime, China) supplemented with a protease inhibitor cocktail (MedChemExpress, USA). The protein concentration of the samples was determined using the BCA protein assay. Equal amounts of total protein were separated by a 10% or 12% SDS-PAGE and transferred to PVDF membranes (Bio-Rad, Hercules, CA, USA). The membrane containing the blotted protein was blocked for 1 hour at room temperature with TBS-T containing 5% skim milk and incubated overnight at 4°C with primary antibodies. After washing with TBS-T, the membrane was incubated for 1 hour at room temperature with an HRP-conjugated secondary antibody. Protein bands were visualized using enhanced chemiluminescence (Thermo Fisher Scientific, Waltham, MA, USA).

The primary antibodies used in these experiments were as follows: anti-CD9 (rabbit, 1:1000, Abcam, ab223052); anti-TSG101 (rabbit, 1:5000, Abcam, ab125011); anti-Calnexin (rabbit, 1:5000, Abcam, ab227310); anti-GOLIM4 (rabbit, 1:5000, Abcam, ab197595); anti-Cleaved Caspase-3 (rabbit, 1:1000, CST, 9664S); anti-BCL-2 (rabbit, 1:1000, CST, 3498S); anti-BAX (rabbit, 1:1000, CST, 2772S); anti-β-actin (mouse, 1:1000, CST, 3700S); anti-PI3K (rabbit, 1:1000, zenbio, 380849); anti-phospho-PI3K (rabbit, 1:1000, zenbio, 310164); anti-AKT (mouse, 1:5000, Proteintech, 60203-2-Ig); and anti-phospho-AKT (mouse, 1:2000, Proteintech, 66444-1-Ig).

### 2.8 Total RNA extraction and qRT-PCR

Total RNA was extracted from processed cells using TRIzol reagent (Takara, Japan), according to the manufacturer’s protocol. Subsequently, 1 μg of total RNA was reverse transcribed into cDNA using the HiScript III 1st Strand cDNA Synthesis Kit (Vazyme, China), as per the provided guidelines. Quantitative real-time PCR (qRT-PCR) was then performed using AceQ qPCR SYBR Green Master Mix (Vazyme, China). The expression levels were detected using an ABI 7500 sequence detection system (Applied Biosystems, CA, USA). The primers used were as follows: miR-202-5p, 5’-UUCCUAUGCAUAUACUUCUUUG-3’; U6, 5’-CTCGCTTCGGCAGCACA-3’; GOLIM4-forward, 5’-AAAGAGACAACCAGCACCAAGAT-3’, reverse, 5’-CACTTGCTCGGCTTCTTCAAATT-3’; β-actin-forward, 5’-CCTTCCTGGGCATGGAGTC-3’, reverse, 5’-TGATCTTCATTGTGCTGGGTG-3’. All experiments were independently repeated three times.

### 2.9 Dual-luciferase reporter assay

The dual-luciferase reporter assay was employed to confirm the interaction between miR-202-5p and GOLIM4. Initially, the 3’ UTR sequence of GOLIM4 was amplified, and cloned into the pmirGLO vector to create the wild-type (GOLIM4-WT) and mutant (GOLIM4-MT) reporter plasmids. Next, 293T cells were cultured to achieve 70%-80% confluence and transfected with one of plasmid combinations: mimics NC + GOLIM4-WT, miR-202-5p mimics + GOLIM4-WT, mimics NC + GOLIM4-MT, or miR-202-5p mimics + GOLIM4-MT. Forty-eight hours post-transfection, the cells were lysed, and the luciferase activities of both firefly and Renilla luciferases were measured using the Dual-Luciferase Reporter Assay System (Promega). Renilla luciferase activity used as an internal control, and the relative luciferase activity was calculated for each group.

### 2.10 Animal model

Male C57BL/6 mice (20–25 g, 8–10 weeks old) obtained from the Animal Experiment Center of Chongqing Medical University were used to establish a renal IRI model. The mice were randomly assigned to the following groups: (a) sham group (n = 6), (b) IR + PBS group (n = 6), and (c) IR + HucMSC-sEVs group (n = 6). Six hours prior to surgery, 100 μL of PBS or 100 μL of HucMSC-sEVs (5 × 10^10^ particles) were injected via the tail vein. The animals were housed in a pathogen-free environment under a controlled 12-hour light/12-hour dark cycle and had ad libitum access to food and water.

The procedure for establishing the renal IRI model in mice was as follows: Mice were anesthetized with an intraperitoneal injection of 1% pentobarbital sodium (50 mg/kg) and placed on a heating pad. The left kidney was excised to prevent compensatory effects, and the right renal pedicle was clamped with a microvascular clamp, causing the kidney to change color from red to purple. The clamp was removed after 35 minutes (30). Mice in the sham surgery group underwent the same surgical procedure, except the renal pedicle was not clamped. One day later, the mice were euthanized, and blood and kidney tissues were collected. The animal research protocol was approved by the Animal Care and Use Committee of Chongqing Medical University.

### 2.11 Assessment of renal function

The collected blood samples were centrifuged at 2000 × g for 15 minutes to obtain serum. The levels of serum creatinine (Scr) and blood urea nitrogen (BUN) were measured by the Biochemical Laboratory of Sichuan Scientist Biotechnology Co., Ltd. (Sichuan, China).

### 2.12 Hematoxylin-eosin staining

The excised kidney tissues were fixed in 4% paraformaldehyde, embedded in paraffin, and sectioned. The paraffin sections were dewaxed in xylene, rehydrated in graded ethanol, and stained with hematoxylin for 10 minutes, followed by rinsing with tap water to halt the staining. The sections were then briefly treated with 0.7% hydrochloric acid-ethanol, washed with tap water to achieve bluing, and stained with eosin for 30 seconds. After dehydration in 95% ethanol, anhydrous ethanol, and clearing in xylene, the sections were air-dried and mounted with neutral resin. Tissue morphology was examined and photographed under an optical microscope.

Renal morphological evaluation was based on criteria including tubular epithelial cell swelling, tubular atrophy and dilation, loss of the brush border, vacuolization, and cast formation (31). The severity of injury was scored according to the percentage of damaged area, with a pathological score ranging from 0 to 5 (0: no damage, 1: < 10% damage, 2: 10-25%, 3: 25-50%, 4: 50-75%, 5: > 75%).

### 2.13 Immunohistochemical staining

Paraffin-embedded kidney sections were dewaxed in xylene, rehydrated in ethanol, and subjected to antigen retrieval in citrate buffer for 30 minutes. Endogenous peroxidase activity was blocked with 3% hydrogen peroxide, and sections were then blocked with normal goat serum for 15 minutes. After overnight incubation with the primary antibody at 4°C, sections were incubated with the secondary antibody at 37°C for 30 minutes, followed by incubation with a streptavidin-horseradish peroxidase complex for 30 minutes. The sections were stained with DAB for 1 minute and counterstained with hematoxylin for 10 seconds. Marker expression was quantified using Image-Pro Plus (IPP, version 6.0). The immunohistochemistry score was evaluated based on the stain intensity and the proportion of positive cells.

### 2.14 Statistical analysis

GraphPad Prism 9 (GraphPad Software, USA) was utilized for the data analysis. The data from at least three independent experiments are presented as mean ± standard deviation (M ± SD). An unpaired two-tailed Student’s t-test was used to evaluate the differences between two groups, and one-way ANOVA was used for the comparisons among multiple groups. A P-value < 0.05 was considered statistically significant.

## 3 Results

### 3.1 Characterization of HucMSC-sEVs

**Figure 1:**
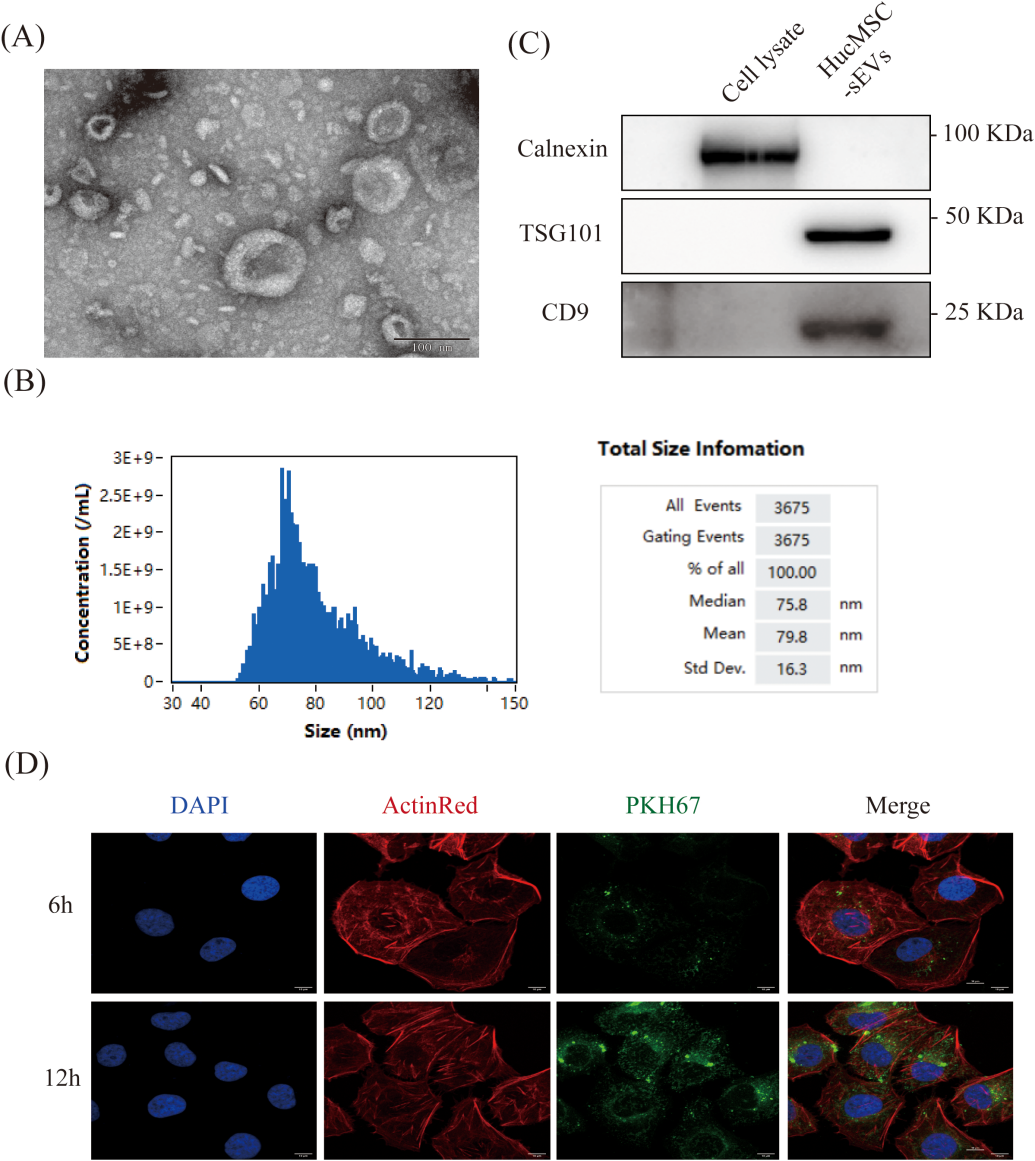
Characterization of HucMSC-sEVs. **(A)** The morphology of HucMSC-sEVs was observed using transmission electron microscopy (TEM, scale bar = 100 nm). **(B)** Western blotting was performed to confirm the protein expression of TSG101, CD9 and Calnexin in HucMSC cell lysate and HucMSC-sEVs. **(C)** Nanoflow cytometry was conducted to reveal the particle size of HucMSC-sEVs. **(D)** *In vitro* fluorescence microscopy images were obtained to show the uptake of PKH67-labeled HucMSC-sEVs by HK-2 cells at 6 and 12 hours (scale bar = 10 μm).

We employed differential ultracentrifugation to isolate sEVs from HucMSC cells (HucMSC-sEVs). To characterize the HucMSC-sEVs, we initially examined them using a TEM, which revealed their characteristic biconcave, rounded morphology, consistent with the established morphological traits of sEVs (**Figure 1A**). NanoFCM analysis further demonstrated that the particle size of HucMSC-sEVs predominantly ranged from 60 to 100 nm, with an average size of 79.8 nm, corroborating their classification as sEVs (28) (**Figure 1B**). The expression of sEVs markers CD9 and TSG101 was evaluated via western blotting, confirming the presence of both proteins in HucMSC-sEVs, while the absence of the endoplasmic reticulum marker Calnexin was also detected, thereby substantiating the high purity of the sEVs (**Figure 1C**). To ascertain the absorption of HucMSC-sEVs by HK-2 cells, we introduced sEVs tagged with PKH67 (green) into the cells and monitored the process using laser scanning confocal microscopy. The outcomes indicated that HK-2 cells, marked with DAPI (blue) and ActinRed (red), initiated the internalization of HucMSC-sEVs within 6 hours post-tagging, with a marked increase in absorption after 12 hours (**Figure 1D**). This suggested that HucMSC-sEVs could be effectively absorbed by HK-2 cells and perform their designated roles. The aforementioned experiments confirmed that we successfully isolated and acquired HucMSC-sEVs.

### 3.2 HucMSC-sEVs suppress OGD/R-induced apoptosis in HK-2 cells

**Figure 2:**
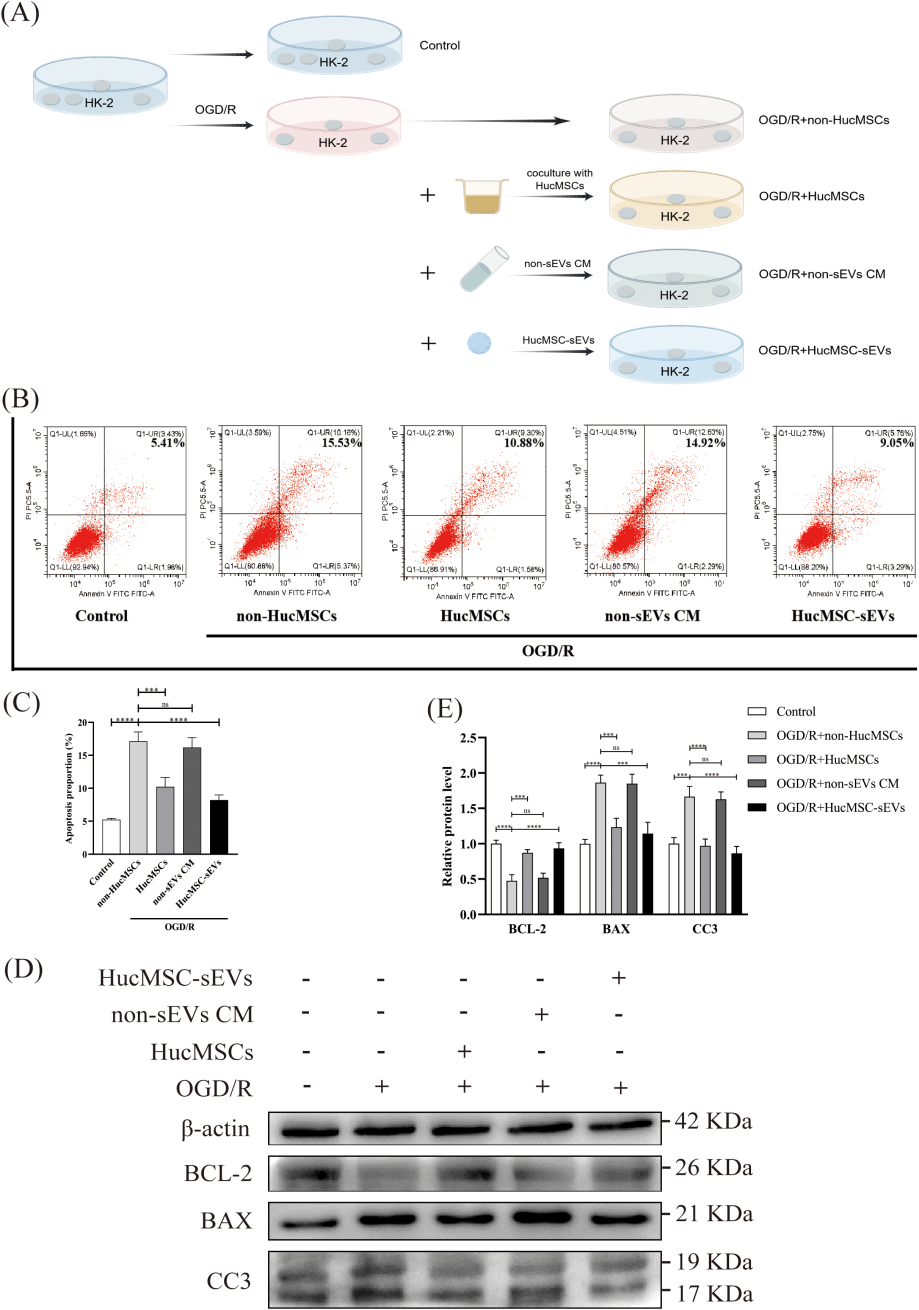
HucMSC-sEVs suppress OGD/R-induced apoptosis in HK-2 cells. **(A)** Schematic diagram illustrating the different treatments applied to HK-2 cells. **(B)** Apoptosis levels in HK-2 cells were assessed by flow cytometry after different treatments. **(C)** The quantitative analyses of flow cytometry assays. **(D)** The expression levels of apoptosis-related proteins (BCL-2, BAX, CC3) were evaluated by Western blotting. **(E)** The quantitative analyses of Western blotting assays; the results were normalized according to the control group. non-HucMSC, the OGD/R-treated HK-2 cells without HucMSC-sEVs or coculture. (n = 3, ***p < 0.001, ****p < 0.0001, ns means no significance).

To conduct a more thorough investigation into the function of HucMSC-sEVs in OGD/R-induced apoptosis of HK-2 cells, as illustrated in **Figure 2A**, we subjected HK-2 cells to various treatments. Subsequently, the apoptosis of cells in each group was evaluated using flow cytometry. OGD/R significantly increased apoptosis in HK-2 cells compared to the control. However, coculture with HucMSCs or direct addition of HucMSC-sEVs reduced apoptosis, with the latter showing a more pronounced effect (**Figures 2B, C**). Additionally, western blotting revealed that the expression of the pro-apoptotic protein Bcl-2 associated X protein (BAX) and the activated apoptosis execution protein Cleaved Caspase-3 (CC3) was notably increased, whereas the expression of the anti-apoptotic protein BCL-2 was significantly reduced in HK-2 cells after OGD/R treatment. Nonetheless, an up-regulation in the expression of the apoptosis inhibitor protein BCL-2 was observed in the co-culture group or the group with HucMSC-sEVs addition, and the expression of both the pro-apoptotic protein BAX and the activated apoptosis execution protein CC3 was suppressed. This further corroborated the significant role of HucMSC-sEVs in alleviating apoptosis (**Figures 2D, E**). In summary, HucMSCs were capable of alleviating OGD/R-induced HK-2 cell injury, and this protective effect was likely mediated by sEVs.

### 3.3 Enrichment of miR-202-5p in HucMSC-sEVs and its protective effect on OGD/R-induced apoptosis in HK-2 cells

**Figure 3:**
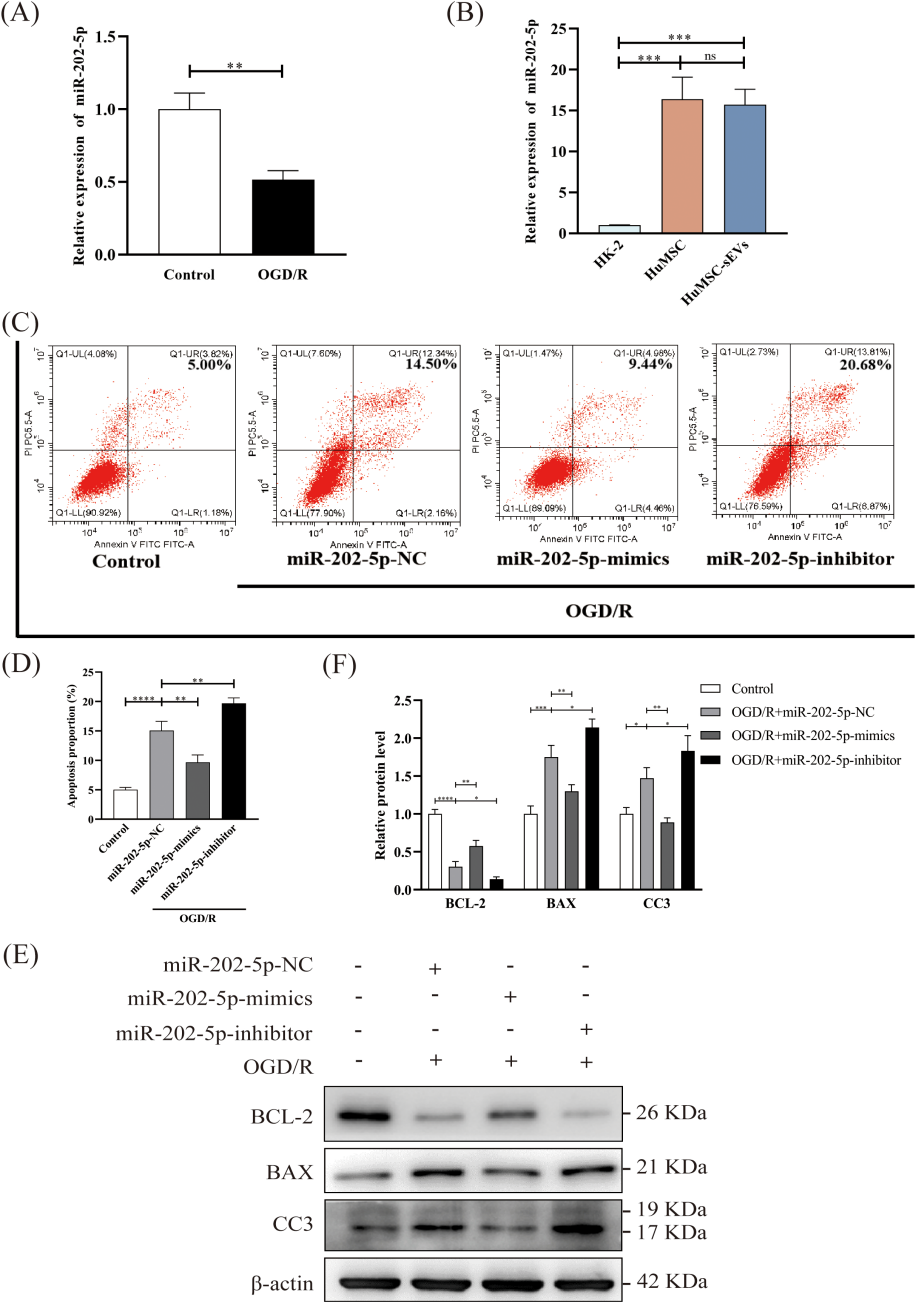
Enrichment of miR-202-5p in HucMSC-sEVs and its protective effect on OGD/R-induced apoptosis in HK-2 cells. **(A, B)** The expression level of miR-202-5p was measured by RT-qPCR in the Control and OGD/R groups, as well as in HK-2 cells, HucMSC cells, and HucMSC-sEVs. **(C)** Apoptosis levels in HK-2 cells treated with OGD/R, along with miR-202-5p mimics or miR-202-5p inhibitor, were analyzed by flow cytometry. **(D)** The quantitative analyses of flow cytometry assays. **(E)** The expression levels of apoptosis-related proteins (BCL-2, BAX, CC3) were evaluated by Western blotting. **(F)** The quantitative analyses of Western blotting assays; the results were normalized according to the control group. (n = 3, *p < 0.05, **p < 0.01, ***p < 0.001, ****p < 0.0001, ns means no significance).

Previous studies have elucidated the function of miR-202-5p in ischemia-reperfusion injury within cardiac and pulmonary tissues, as well as its prevalence in bone marrow mesenchymal stem cells (32–34). However, its role in renal IRI and HucMSCs has not been previously explored. Consequently, we initially employed quantitative reverse transcription polymerase chain reaction (qRT-PCR) to ascertain its expression levels. The findings indicated a significant reduction in miR-202-5p expression in HK-2 cells subsequent to OGD/R treatment compared to the control group (**Figure 3A**). Additionally, the expression of miR-202-5p was notably elevated in both HucMSC cells and HucMSC-sEVs compared to HK-2 cells (**Figure 3B**). Further analysis via flow cytometry revealed that miR-202-5p-mimics exacerbated apoptosis induced by OGD/R, whereas the miR-202-5p-inhibitor intensified it (**Figures 3C, D**). Western blotting demonstrated that miR-202-5p-mimics suppressed the down-regulation of BCL-2 expression and the up-regulation of BAX and CC3 expression caused by OGD/R, suggesting that miR-202-5p exerts a protective effect by modulating the expression of proteins associated with apoptosis (**Figures 3E, F**). Collectively, these experimental outcomes indicate that miR-202-5p within HucMSC-sEVs plays a crucial role in impeding OGD/R-induced apoptosis in HK-2 cells.

### 3.4 Targeting of GOLIM4 by miR-202-5p modulates activity of the PI3K/AKT pathway

**Figure 4:**
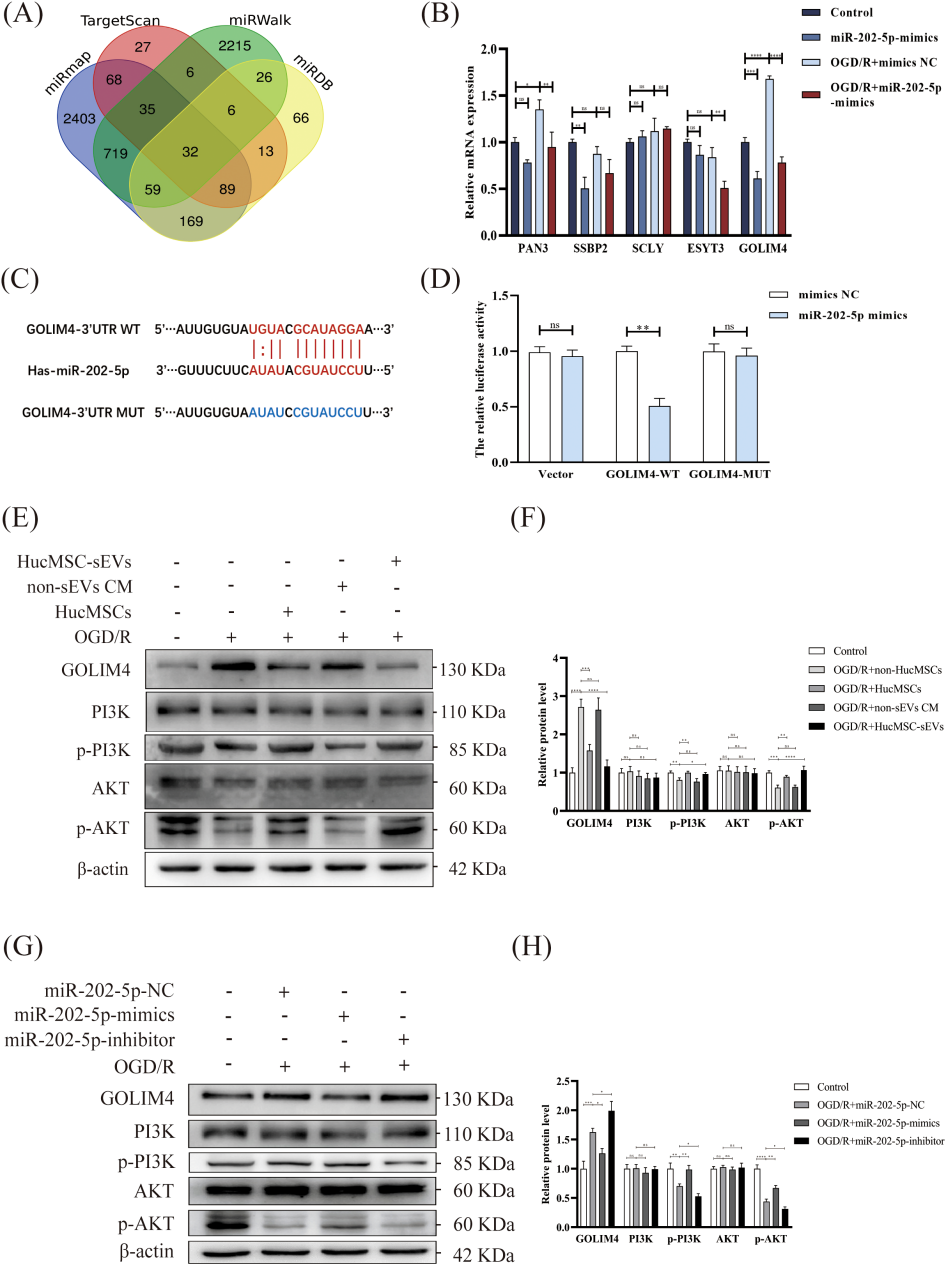
Targeting of GOLIM4 by miR-202-5p modulates activity of the PI3K/AKT pathway. **(A)** Venn diagram showing the potential targets of miR-202-5p predicted by four online prediction sites (miRmap, Targetscan, miRWalk, miRDB). **(B)** The expression levels of potential targets were measured by RT-qPCR in HK-2 cells that were either treated with or without OGD/R and transfected with or without miR-202-5p. **(C)** The predicted binding sites between miR-202-5p and GOLIM4 were identified using TargetScan. **(D)** A dual-luciferase reporter assay was performed to validate the interaction between miR-202-5p and GOLIM4. **(E-H)** The expression of GOLIM4 and PI3K/AKT pathway related proteins in the different treatment groups was examined by western blotting, and the quantitative analyses; the results were normalized according to the control group. (n = 3, *p < 0.05, **p < 0.01, ***p < 0.001, ****p < 0.0001, ns means no significance).

In order to investigate the function of miR-202-5p, we initially utilized four online bioinformatics tools (TargetScan, miRWalk, miRmap, miRDB) to forecast the potential target genes of miR-202-5p. The Venn diagrams indicated that 32 target genes were situated within the intersection region of the prediction outcomes from these tools (**Figure 4A**). Subsequent experimental results demonstrated that the expression of GOLIM4 was notably diminished in the miR-202-5p-mimics group in the presence of OGD/R treatment or not, whereas the expression changes of the other target genes were not pronounced (**Figure 4B**). Furthermore, dual luciferase reporter assay confirmed that miR-202-5p could bind to the 3’UTR region of GOLIM4, thereby affirming that GOLIM4 is indeed a target gene of miR-202-5p (**Figures 4C, D**). Additionally, we assessed proteins associated with the PI3K/AKT signaling pathway, which are integral to cell survival. Western blotting revealed that in HK-2 cells treated with OGD/R, the phosphorylation levels of proteins related to the PI3K/AKT signaling pathway (PI3K and AKT) significantly decreased. However, it is noteworthy that after treatment with HucMSC-sEVs or miR-202-5p-mimics, the phosphorylation levels of these proteins were notably restored, whereas the miR-202-5p-inhibitor yielded the converse effect (**Figures 4E–H**). Based on these findings, we conclude that miR-202-5p within HucMSC-sEVs safeguards HK-2 cells against OGD/R-induced injury by specifically binding to and inhibiting the expression of GOLIM4 while simultaneously activating the PI3K/AKT pathway.

### 3.5 GOLIM4 silencing rescues miR-202-5p inhibitor-induced apoptosis and PI3K/AKT pathway dysfunction in injured HK-2 cells

**Figure 5:**
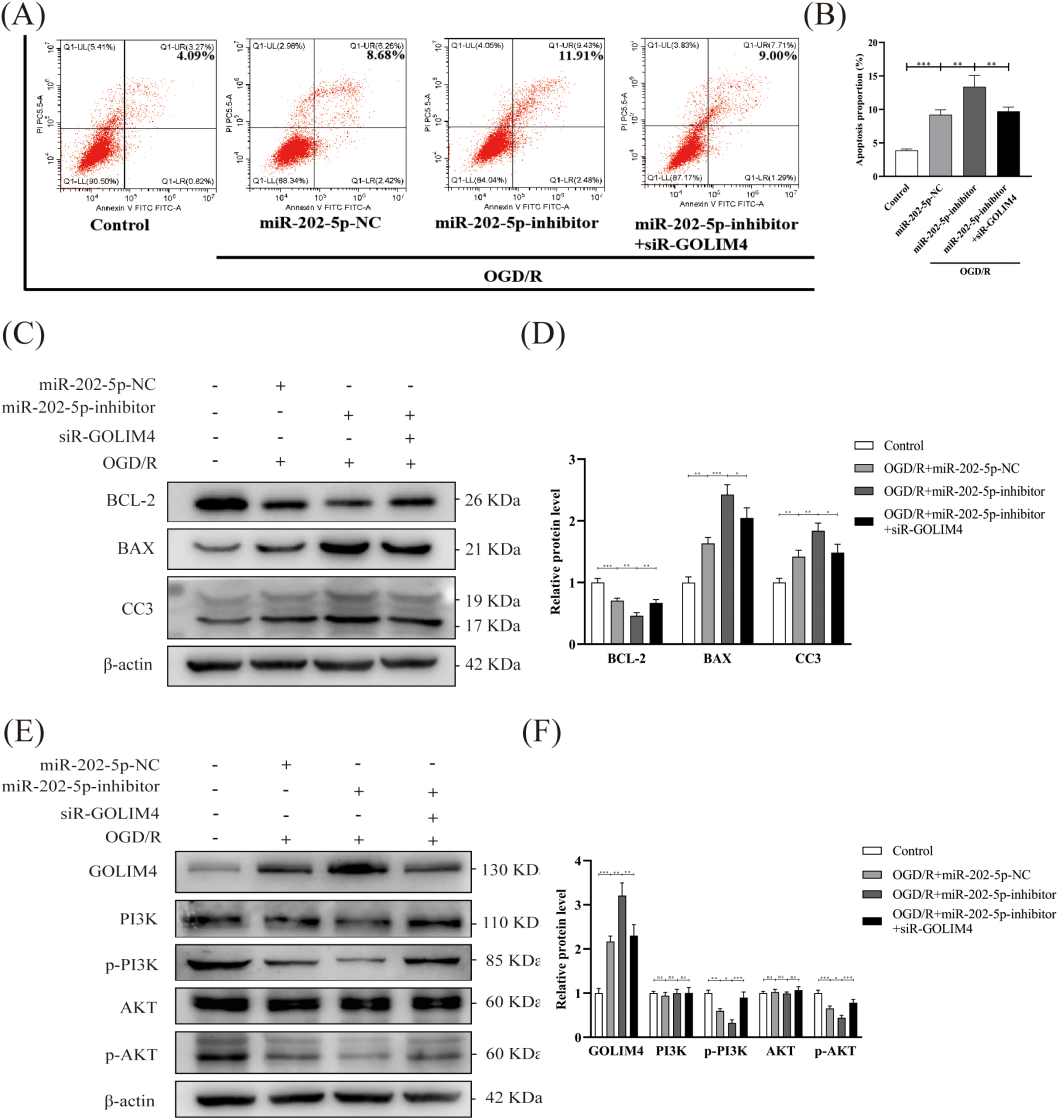
GOLIM4 silencing rescues miR-202-5p inhibitor-induced apoptosis and PI3K/AKT pathway dysfunction in injured HK-2 cells. **(A, B)** Apoptosis levels in HK-2 cells were evaluated by flow cytometry after transfection with or without siR-GOLIM4, and the quantitative analyses. **(C-F)** The expression levels of apoptosis-related proteins, GOLIM4, and PI3K/AKT pathway-related proteins were examined by Western blotting in HK-2 cells transfected with or without siR-GOLIM4, and the quantitative analyses; the results were normalized according to the control group. (n = 3, *p < 0.05, **p < 0.01, ***p < 0.001, ns means no significance).

To validate the aforementioned findings, rescue experiments were conducted. Initially, flow cytometry revealed that the silencing of GOLIM4 notably diminished the exacerbation of apoptosis induced by the miR-202-5p inhibitor in the context of OGD/R treatment, indicating that GOLIM4 silencing could partially counteract the effects of the miR-202-5p inhibitor (**Figures 5A, B**). Subsequently, Western blotting further confirmed that GOLIM4 silencing restored of BCL-2 expression and the suppression of BAX and CC3 expression, simultaneously reinstating the phosphorylation levels of PI3K and AKT within the PI3K/AKT pathway (**Figures 5C–F**). These outcomes further elucidated the pivotal role of miR-202-5p in HucMSC-sEVs’ protection of HK-2 cells against OGD/R injury, mediated through the GOLIM4/PI3K/AKT axis.

### 3.6 HucMSC-sEVs attenuate IR-induced kidney injury *in vivo*

**Figure 6:**
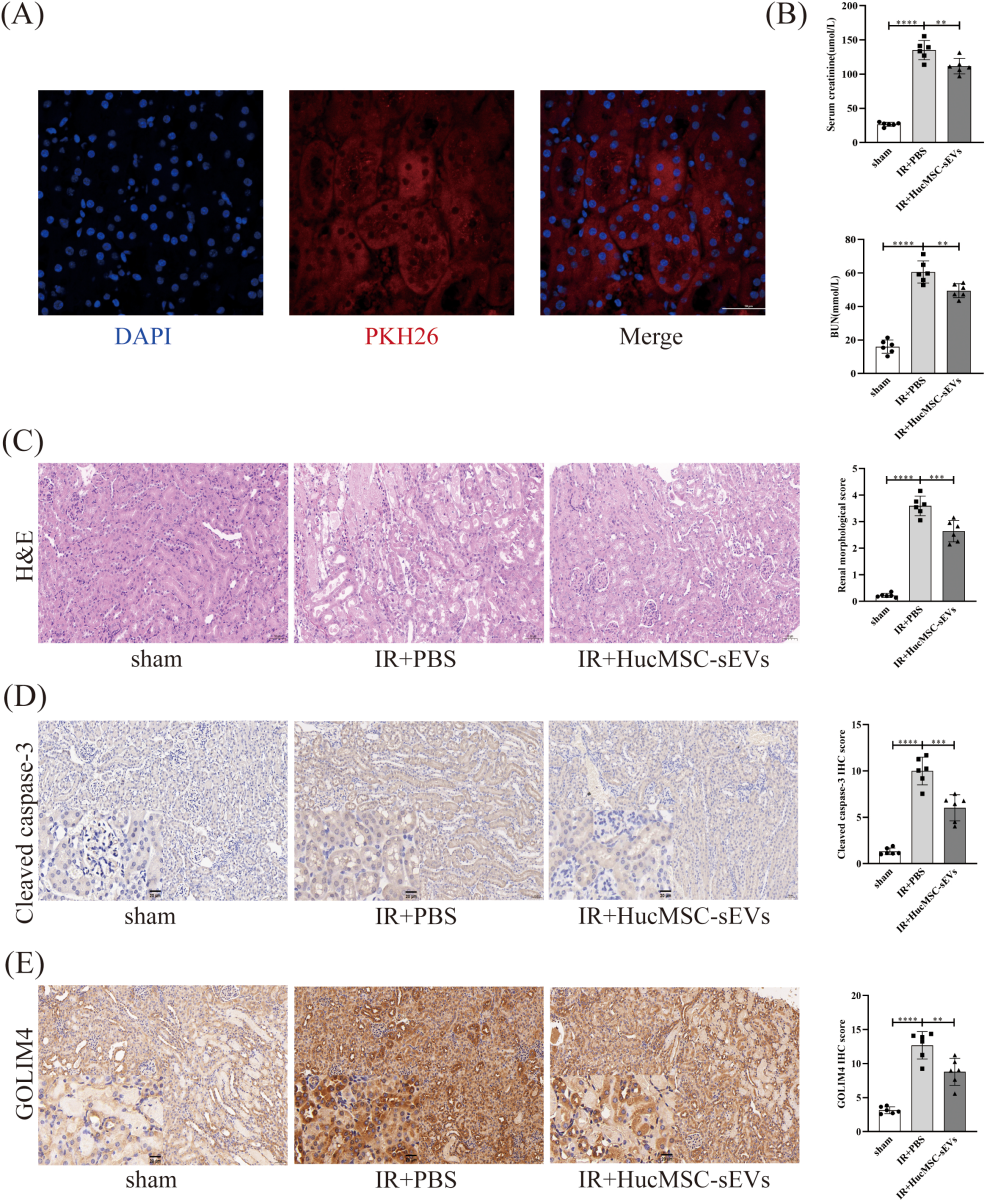
HucMSC-sEVs attenuate IR-induced kidney injury *in vivo*. **(A)** Immunofluorescence showing the uptake of PKH26-labeled HucMSC-sEVs by kidney cells (Scale bar = 50 μm). **(B)** The levels of serum creatinine (SCr) and blood urea nitrogen (BUN) were measured in the sham, IR+PBS, and IR+HucMSC-sEVs groups. **(C)** The degree of kidney damage in different treatment groups is illustrated by hematoxylin-eosin staining (Scale bar = 50 μm). **(D, E)** Immunohistochemical staining was performed to assess the expression levels of cleaved caspase-3 and GOLIM4 in kidney tissues from the various treatment groups (Scale bar = 50 μm), with magnified insets (20 μm) in the lower left corner of each image to provide a closer view of the staining. (n = 6, **p < 0.05, ***p < 0.001, ****p < 0.0001).

To further investigate the function of HucMSC-sEVs *in vivo*, we established a mouse model of renal IRI. Immunofluorescence analysis indicated that HucMSC-sEVs, labeled with PKH26 (red), were effectively absorbed by mouse kidney cells, thereby affirming the delivery and functionality of HucMSC-sEVs within the organism (**Figure 6A**). Biochemical analysis results demonstrated that Scr and BUN levels in mice subjected to IR+PBS were markedly elevated compared to those in the sham group. Conversely, Scr and BUN levels in mice treated with HucMSC-sEVs were significantly reduced relative to the IR+PBS group, indicating that HucMSC-sEVs can alleviate renal damage (**Figure 6B**). Histopathological examination via HE staining revealed that HucMSC-sEVs treatment notably alleviated renal tissue injury caused by IR (**Figure 6C**). Immunohistochemical staining further substantiated that HucMSC-sEVs treatment mitigated the increased expression of CC3 and GOLIM4 induced by IR (**Figures 6D, E**). These animal experiments corroborated the protective role of HucMSC-sEVs in renal IRI.

## 4 Discussion

IRI stands as a principal pathological process underlying AKI, markedly disrupting renal function and resulting in increased mortality rates (6, 35). Currently, effective treatments for IRI remain limited (5). In the present study, we demonstrate that HucMSC-sEVs exert a protective effect on renal tubular epithelial cells against IRI *in vitro* and *in vivo*. Notably, we elucidate a novel mechanism through which HucMSC-sEVs deliver miR-202-5p to renal cells, leading to the suppression of GOLIM4 expression and subsequent modulation of the PI3K/AKT signaling pathway. This interaction reduces renal tubular cell apoptosis, thereby mitigating renal injury induced by IRI. These findings enhance our understanding of the protective role of HucMSC-sEVs in renal IRI and offer a promising therapeutic strategy for managing AKI through the application of HucMSC-sEVs.

In recent years, mesenchymal stem cells (MSCs) have garnered extensive research attention for their potential to mitigate organ damage through the secretion of sEVs that deliver miRNAs (36–38). For instance, Kim et al. (39) demonstrated that sEVs from tonsil-derived MSCs can alleviate the activation of hepatic stellate cells and liver fibrosis. Lee et al. (40) indicated that sEVs from MSCs protect cardiomyocytes from doxorubicin-induced cardiomyopathy by upregulating the expression of survivin. Additionally, MSC-sEVs have been found to regulate processes such as the cell cycle and apoptosis by delivering miRNAs like miR-125b-5p and miR-26a-5p, thereby reducing renal injury caused by IRI (41, 42). These studies support our findings, emphasizing the pivotal role of miRNAs in the renal repair process mediated by MSC-sEVs. Nevertheless, the novelty of our research lies in the discovery that miR-202-5p, delivered by HucMSC-sEVs, targets GOLIM4 to regulate the PI3K/AKT signaling pathway and reduce renal tubular cell apoptosis, offering new molecular targets and therapeutic insights.

In this study, we found that HucMSC-sEVs deliver miR-202-5p into renal tubular cells, suppressing GOLIM4 expression and modulating the PI3K/AKT pathway. The PI3K/AKT pathway plays a crucial role in cell survival, proliferation, and apoptosis is closely associated with the development and progression of various renal diseases (8, 43, 44). For example, Liu et al. (8) found that inhibition of Brd4 blocked renal apoptosis and the expression of ERS proteins by preventing the production of FoxO4-dependent ROS through the PI3K/AKT pathway. GOLIM4, a protein involved in the intracellular membrane transport system, has not been extensively studied in the context of IRI (24). Our findings revealed that the suppression of GOLIM4 significantly reduced renal tubular cell apoptosis, thereby alleviating renal injury caused by IRI. This discovery not only uncovers a novel mechanism by which miR-202-5p regulates renal injury but also provides new theoretical evidence for further exploration of miRNA-based therapies for AKI. Additionally, HucMSC-sEVs contain a variety of bioactive molecules, including miRNAs and proteins, which can exert diverse effects on target cells (12, 19, 45, 46). MiR-202-5p, through the regulation of numerous target genes, may extend beyond GOLIM4 and could potentially contribute to the repair of renal injury via other target genes. Thus, HucMSC-sEVs demonstrate extensive biological effects and possess the potential to offer supplementary therapeutic targets for forthcoming precision medicine initiatives.

Despite the valuable insights into the protective effects of HucMSC-sEVs against renal IRI, several limitations remain. Firstly, while the efficacy of HucMSC-sEVs has been validated *in vitro* and *in vivo*, their applicability to clinical settings requires further investigation. Future research should involve more comprehensive animal studies and validation using clinical specimens to assess the efficacy and safety profiles of HucMSC-sEVs across a broader range of individuals. Secondly, although the role of GOLIM4 within the PI3K/AKT pathway has been clarified, the precise mechanisms by which GOLIM4, as an emerging regulatory factor, contributes to renal injury are yet to be fully elucidated. Future studies should define the specific functions of GOLIM4 and its interactions with other molecular components. Additionally, the methodologies for extracting and purifying HucMSC-sEVs, as well as the therapeutic differences among HucMSC-sEVs derived from various sources, need further examination.

Future research could be directed in several promising directions. First, studies should investigate the expression of miR-202-5p in clinical AKI samples to assess its potential as a biomarker. Advances in molecular biology could position miRNAs as powerful tools for early AKI diagnosis and prognosis. Second, further preclinical studies should be conducted to explore the therapeutic effects of HucMSC-sEVs derived from various sources and processed under different conditions, as well as their potential for integration with other therapeutic strategies, like pharmacological treatments or gene therapy. Moreover, with the rapid progress of cell and gene therapies, HucMSC-sEVs, acting as natural delivery vehicles for multiple therapeutic molecules, hold significant potential for practical application.

## 5 Conclusion

In conclusion, this study demonstrates that HucMSC-sEVs play a significant role in alleviating renal IRI both *in vitro* and *in vivo*. The abundant miR-202-5p in HucMSC-sEVs exerts a protective effect by targeting GOLIM4 and activating the PI3K/AKT signaling pathway. These findings suggest that HucMSC-sEVs and miR-202-5p content as potential therapeutic targets for the treatment of renal IRI.

## Data Availability

The original contributions presented in the study are included in the article/supplementary material. Further inquiries can be directed to the corresponding author.
